# Effects of Essential Oils of *Elettaria cardamomum* Grown in India and Guatemala on Gram-Negative Bacteria and Gastrointestinal Disorders

**DOI:** 10.3390/molecules26092546

**Published:** 2021-04-27

**Authors:** Aftab Alam, Najeeb Ur Rehman, Mohd Nazam Ansari, Amber Hanif Palla

**Affiliations:** 1 Department of Pharmacognosy, College of Pharmacy, Prince Sattam Bin Abdulaziz University, Al-Kharj 11942, Saudi Arabia; 2 Department of Pharmacology and Toxicology, College of Pharmacy, Prince Sattam Bin Abdulaziz University, Al-Kharj 11942, Saudi Arabia; n_rehman5@hotmail.com (N.U.R.); nazam.ansari@gmail.com (M.N.A.); 3 Department of Basic Medical Sciences (Pharmacology), Salim Habib University, Deh Dih, Korangi Creek, Karachi 74900, Pakistan; amber.palla@shu.edu.pk

**Keywords:** *Elettaria cardamomum*, GC-MS, essential oil, antibacterial, antidiarrheal, antispasmodic

## Abstract

The present study examined the chemical composition and antimicrobial and gastrointestinal activity of the essential oils of *Elettaria cardamomum* (L.) Maton harvested in India (EC-I) and Guatemala (EC-G). Monoterpenes were present in higher concentration in EC-I (83.24%) than in EC-G (73.03%), whereas sesquiterpenes were present in a higher concentration in EC-G (18.35%) than in EC-I (9.27%). Minimum inhibitory concentrations (MICs) of 0.5 and 0.25 mg/mL were demonstrated against *Pseudomonas aeruginosa* in EC-G and EC-I, respectively, whereas MICs of 1 and 0.5 mg/mL were demonstrated against *Escherichia coli* in EC-G and EC-I, respectively. The treatment with control had the highest kill-time potential, whereas the treatment with oils had shorter kill-time. EC-I was observed to be more potent in the castor oil-induced diarrhea model than EC-G. At 100 and 200 mg/kg, P.O., EC-I exhibited 40% and 80% protection, respectively, and EC-G exhibited 20% and 60% protection, respectively, in mice, whereas loperamide (10 mg/kg, i.p., positive control) exhibited 100% protection. In the in vitro experiments, EC-I inhibited both carbachol (CCh, 1 µM) and high K^+^ (80 mM)-induced contractions at significantly lower concentrations than EC-G. Thus, EC-I significantly inhibited *P. aeruginosa* and *E. coli* and exhibited more potent antidiarrheal and antispasmodic effects than EC-G.

## 1. Introduction

*E. cardamomum* (L.) Maton, belonging to the *Zingiberaceae* family (local name: cardamom), is an expensive and commercially significant spice that is in demand worldwide. Although it is native to India and Sri Lanka, it is also grown in Guatemala, Thailand, El Salvador, Malay Archipelago, Vietnam, Papua New Guinea, Cambodia, Laos, and Tanzania, with Guatemala being the largest producer of *E. cardamomum* in the world [1,2].

Volatile oils were demonstrated to be good sources of bioactive compounds, in the form of cyclic and acyclic, non-oxygenated or oxygenated hydrocarbons of monoterpenes, sesquiterpenes, and diterpenes. It is used in the treatment of several disease conditions such relieving pain, wound, nausea, and cancer lesions in folk medicine [3]. The composition of essential oil depends on the family, genus, species, and chemotype of a plant, as well as from the material which it is obtained, growing conditions, harvesting season and geographical origin [4]. Biological activity of *E. cardamomum* essential oil is strictly linked to their chemical composition such as ester α-terpinyl acetate and monoterpene 1,8-cineole, α-terpineol, linalool, α-pinene, and several others [5]. International organisation for standardization (ISO) developed ISO 4733:2004 oil of cardamom [*Elettaria cardamomum* (L.) Maton] standard; the data clearly reflect the percentage of the major components of the essential oil in different batches of seeds [6]. A wide range of aromatic plants or its parts have been explored for the presence of volatile compounds and evaluated for the antibacterial, antifungal, and antiviral effects [7]. Owing to their biological activities and distinctive flavor and fragrance possessions, essential oils have excellent business potential on the global market due to high demands in cosmetics, pharmaceuticals, food products industries, and traditional medicine [8,9]. Not only cardamom oil, but also its extracts have promising potential for being used as preservatives in the food industry, owing to their antibacterial and flavoring properties, and are considered as preferred alternative to synthetic compounds [10].

*E. cardamomum* oil is known for its characteristic aroma and is widely used in the food and cosmetic industries as a flavoring and fragrance agent. It is an intestinal smooth muscle relaxant [11] and has exhibited antispasmodic, antidiarrheal, and antibacterial activities [12,13]. The antibacterial effect of essential oil of *E. cardamomum* against several Gram-negative bacteria [14] such as *Escherichia coli* and *Pseudomonas aeruginosa* has been reported. *E. coli* commonly resides in human colon and frequently causes diarrhea [15], whereas *P. aeruginosa* also causes diarrhea, and it may effect immune-deficient or antibiotic-treated individuals, which is difficult to treat due to its innate resistance to several antibiotics [16,17,18]. *P. aeruginosa* transmitted through the food chain may cause gastrointestinal infections [19]. Hence, both of these organisms directly or indirectly are potentially involved in the etiology of diarrhea.

Contradictory reports exist on the efficacy of *E. cardamomum* against both these bacteria. Few studies have reported its efficacy against *E. coli* but not against *P. aeruginosa* [20], whereas another study has reported its efficacy against *P. aeruginosa*, but not against *E. coli* [21]. 

These differences could be attributed to the variability or differences in solvent extraction. Therefore, comparative studies of different species must be conducted not only to understand their full potential as herbs but also to identify the most preferable species, because cardamom growing in a specific region provides more health benefits than those growing in other regions. Additionally, a number of these activities have been tested in aqueous methanolic extracts rather than essential oils, which may exhibit different activities depending on the constituents eluted. Therefore, the present study attempted to compare the chemical composition and antimicrobial activity of the essential oil of Guatemalan and Indian *E. cardamomum* against *E. coli* and *P. aeruginosa* and to explore the in vivo antidiarrheal effect and in vitro antispasmodic activity of the two oils to clarify differences in their medicinal properties.

## 2. Results

### 2.1. Compositions of the Essential Oils

**Figure 1:**
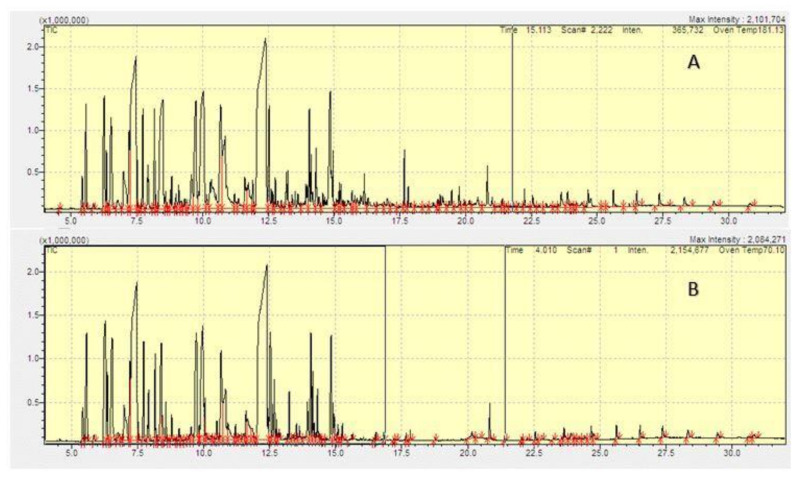
GC-MS chromatograms of *Elettaria cardamomum* of (**A**) (Indian, EC-I) and (**B**) Guatemala, EC-G) essential oils.

Table 1 presents the compositions of the essential oils of EC-I and EC-G capsules identified through GC–MS. About 52 and 63 constituents were identified in the essential oils obtained from EC-I and EC-G, respectively. Total ion–current chromatograms of the typical essential oil (EC-I and EC-G) are shown in Figure 1.

The percentage yield of essential oil extracted from EC-I capsules (4.8%) was higher than EC-G (3.9%) capsules. The oxygenated monoterpene and α-terpinyl acetate were the main constituents of the essential oils of EC-I (24.65%) and EC-G (18.71%), respectively, whereas 1,8 cineole was identified as the second main volatile constituent, with percentages of 14.03% and 10.59%, respectively, in EC-I and EC-G essential oils. Phellandrene, β-pinene, limonene, α-terpinene, ocimene, linalool, terpinen-4-ol, β-fenchyl alcohol, cis-geranyl acetate, guaiene, and nerolidol were identified as other components common to both the samples.

Volatile components were divided into monoterpenes (hydrocarbons and oxygenated), sesquiterpenes (hydrocarbons and oxygenated), diterpenes (hydrocarbons and oxygenated), and non-terpenes, on the basis of their functional groups (Table 2). Out of the total components, approximately 73.03% and 83.24% monoterpenes were identified in the essential oil of EC-G and EC-I capsules, respectively.

Among monoterpenes, approximately 56.87% and 60.22% of oxygenated monoterpenes were identified in the EC-G and EC-I oils, respectively, whereas 16.16% and 21.06% of monoterpene hydrocarbons were identified in the EC-G and EC-I essential oils, respectively. This analysis represented the chemical difference in the EC-G and EC-I samples.

### 2.2. Antimicrobial Activity

The antibacterial activity of EC-I and EC-G is presented in terms of zone of inhibitions diameters (ZOI, mm) and MIC in Table 3.

The ZOI differed marginally with different capsules and microorganisms used in the assay. Both the samples and the standard drug were detected to be inhibitory to *P. aeruginosa* and *E. coli*, and the EC-I oil was showed to be the most active agent. The MIC of EC-G oil was observed to be 0.5 and 1 mg/mL, whereas that of EC-I was 0.25 and 0.5 mg/mL against *P. aeruginosa* and *E. coli* respectively. Thus, the EC-I oil was more active against both the Gram-negative bacteria.

### 2.3. Time-Kill Kinetic Assay

**Figure 2:**
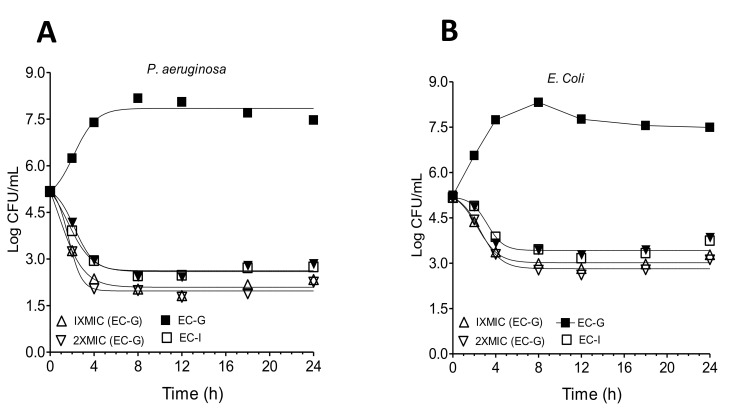
Time-kill analysis of (**A**) *P. aeruginosa* and (**B**) *E. coli*.

Time-kill assays were performed to explore the cell viability (kill-time) of EC-G and EC-I essential oil, and the results were articulated as a logarithm of viable counts (Figure 2). Non-treated *E. coli* exhibited growth from 5.24 to 8.32 log_10_ CFU/mL and moved into the static phase after 8 h. After treatment with EC-G, *E. coli* growth decreased dramatically in the first 8 h and retained steadily at approximately 3.45 × log_10_ CFU/mL, whereas EC-I treatment decreased the growth in the first 8 h and retained steadily at approximately 2.99 × log_10_ CFU/mL, suggesting a stronger EC-I killing efficacy against *E. coli*.

Similarly, non-treated *P. aeruginosa* exhibited growth from 5.17 to 8.17 log_10_ CFU/mL and moved after 8 h into the static phase. After treatment with EC-G, *P. aeruginosa* growth decreased dramatically in the first 4 h and retained steadily at approximately 2.94 × log_10_ CFU/mL. After treatment with EC-I, *P. aeruginosa* growth decreased in the first 4 h and was retained steadily at approximately 2.04 × log_10_ CFU/mL, suggesting a stronger EC-I killing efficacy against *P. aeruginosa*. The plot of both the samples assessed at the 2 × MIC level was almost similar to that at 1 × MIC. The results indicated that EC-G exhibits a lethal effect on *P. aeruginosa* and *E. coli* after 4 h and 8 h, respectively.

Similarly, EC-I exhibited a lethal effect on the growth of both *P. aeruginosa* and *E. coli* after 8 h of incubation. The plot of both samples measured at the 2-MIC stage was approximately identical to that at 1-MIC. EC-I exhibited a rapid killing effect on *P. aeruginosa* development, with a lethal effect after 4 h of incubation and after 8 h on *E. coli*. The effects of EC-I on *P. aeruginosa* and *E. coli* growth were destroyed after 8 h of incubation.

### 2.4. Gastrointestinal Activity

#### 2.4.1. In Vivo Antidiarrheal Study on Mice

Protection in castor oil-provoked diarrhea: Both orally administered samples of EC-I and EC-G exhibited dose-dependent protection of mice, whereas the saline group did not exhibit any effect. At the lower tested dose of EC.I (100 mg/kg), two out of five mice exhibited protection, indicating 40% protection. A higher dose of 200 mg/kg exhibited 80% protection, whereas 20% and 60% protection was observed at lower (100 mg/kg) and higher doses (200 mg/kg), respectively. No diarrheal spot was observed in any mice treated with loperamide (100% protection) (Table 4).

#### 2.4.2. Gut Inhibitory Effects

**Figure 3:**
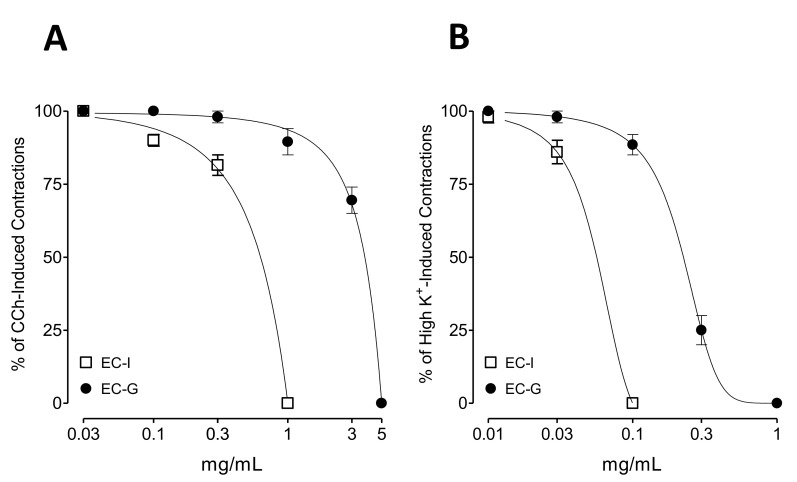
Concentration-response curves showing comparison of the extracted essential oil of *Elettaria cardamomum* of Indian (EC-I) and Guatemala (EC-G) for the inhibitory effect against (**A**) carbachol (CCh, 1 µM) and (**B**) high K^+^-induced contractions in isolated rat ileum preparations. Values shown are mean ± SEM, *n* = 4.

When tested against CCh and high K^+^-mediated spasm in rat ileum preparations, EC-I and EC-G caused dose-dependent (0.01–5 mg/mL) complete inhibition. In CCh-mediated contractions, EC-I exhibited inhibition with resultant EC_50_ values of 0.76 mg/mL [0.54–0.92, 95% confidence interval (CI), *n* = 4], whereas EC-G exhibited inhibition with higher EC_50_ value of 4.22 mg/mL (3.86–4.12, 95% CI, *n* = 4) (Figure 3A). EC-I and EC-G exhibited inhibition against high K^+^-mediated contractions with EC_50_ values of 0.08 mg/mL (0.06–0.09, 95% CI, *n* = 4) and 0.24 mg/mL (0.18–0.28, 95% CI, *n* = 4), respectively (Figure 3B).

## 3. Discussion

Studies have reported that for better fragrances, α-terpinyl acetate is always present in higher amount than 1,8 cineole, which may also be an indicator of high-quality *E. cardamomum* essential oils. In the present study, findings of a higher percentage of α-terpinyl acetate than 1,8 cineole indicated both samples have good quality essential oil similar to earlier reports [22,23].

In this report, monoterpene components such as β-phellandrene, β-pinene, DL-limonene, β-*cis*-ocimene, γ-terpinen, α-terpinene, sabinene, α-phellandrene, camphene, β-fenchyl alcohol, terpinen-4-ol, α-terpinyl acetate, *cis*-geranyl acetate, and D-germacrene were observed to be in higher concentrations in the EC-I essential oil. The content of constituents such as β-trans-ocimene and (+)-2-carene, linalool, Z-citral, trans-geraniol, and (*E*)-ocimenyl acetate was higher in the EC-G essential oil. These components have also been reported by several investigators [24,25,26]. *Trans*-sabinenhydrate was the major oxygenated monoterpenes identified only in the EC-I sample, whereas thymol, α-phellandren-8-ol, (d)-verbenone, and dihydrocarveol were other major oxygenated monoterpene identified only in EC-G.

The concentration of sesquiterpene D-germacrene was higher in the essential oils of EC-I, whereas β-elemene, α-murolene, bicyclo[5.2.0]nonane, 2-methylene-4,8,8-trimethyl-4-vinyl-, δ-guaiene, γ-gurjunene, d-germacrene, *D*-nerolidol, (*Z*,*Z*)-farnesal, trans-caryophyllene oxide, aromadendrene oxide-(1), and farnesyl acetate were in higher concentrations in the essential oil of EC-G capsules. Sesquiterpenes such as α-selinene, α -caryophyllene, and (*Z*,*E*)-farnesal were identified only in the essential oil of EC-I, whereas ledene, alloaromadendrene, α-cadinol, γ-eudesmol, 10-epi, β-spathulenol, longifolenaldehyde, costunolide, and isoaromadendrene epoxide were identified only in EC-G. Diterpene and α-springene were observed in both the samples (0.43% and 1.08% in EC-G and EC-I oils, respectively), whereas cembrene, kauran-18-al, 17-(acetyloxy)-, (4.beta), and thunbergol were found only in the essential oil of EC-G. Some of these components have not been previously reported in the GC-MS analysis of cardamom essential oil.

In the current study, monoterpenes were in higher concentrations in EC-I (83.24%) than in EC-G (73.03%), whereas sesquiterpenes were in higher concentrations in EC-G (18.35%) than in EC-I (9.27%). However, no significant differences in diterpenes (1.03% and 1.08% in the EC-G and EC-I, respectively) were reported between EC-I and EC-G. Gradinaru et al. reported 84.54% oxygenated monoterpenes and 8.27% monoterpene hydrocarbons [27], whereas Kumar et al. reported approximately 87% oxygenated monoterpenes and 8.24% monoterpene hydrocarbons in the essential oil of different cardamom samples [28]. Noumi et al. reported the presence of approximately 88.7% oxygenated monoterpenes and 7% monoterpene hydrocarbons in cardamom essential oils [26].

In the present investigation, two Gram-negative bacterial strains, *P. aeruginosa* and *E. coli*, were chosen for measuring the antibacterial activity as these bacteria are becoming resistant to various drugs, and scientists are exploring new molecules to combat these resistant strains. In this study, both samples exhibited antibacterial effects against both selected Gram-negative bacteria, where the MIC of EC-I was lower than that of EC-G oil. In the current study, we obtained 10.13 and 14.4 mm zone of inhibition and MIC 1 mg/mL and 0.5 mg/mL against *E. coli* for EC-G and EC-I respectively. Similarly, we obtained 12.33 and 16.66 mm zone of inhibition and MIC 0.5 mg/mL and 0.25 mg/mL against *P. aeruginosa* for EC-G and EC-I respectively, which indicates that the tested oils are effective. In a previous study, Noumi et al., (2018) reported the range of MIC of *E. coli* (0.048–0.097mg/mL) and *P. aeruginosa* (0.048 mg/mL) for the green cardamom essential oil, which supports the present antimicrobial activity [26]. Although we tested the zone of inhibition for gentamycin against *P. aeruginosa* and *E. coli* (22.7 mm and 19.67 mm, respectively), we could not test the MIC due to certain limitations. Similar zones of inhibition for gentamycin have also been reported in previous studies [29,30]. Compared to the MIC of gentamycin from literature, i.e., less than 0.00156 mg/mL and 0.00313 mg/mL against *E. coli* and *P. aeruginosa,* respectively [31], we get another interpretation, which is that the antibacterial activity is present, but compared to the positive control, it seems marginal. This, however, is not surprising as an extract is the mixture of compounds with different chemical constituents amongst whom some may and some may not have antibacterial activities. Hence, isolation of these compounds would be the ideal method to predict whether or not the antibacterial activity is at an appreciable extent or not. Thus, for adding further validity, we will direct our future studies to not only assess the effect of cardamom oil on different pathogenic bacteria involved in gastrointestinal diseases but we will also test the different compounds isolated and subsequently compare them with respective controls including vancomycin and gentamycin for Gram-positive and Gram-negative microbes respectively.

The major compounds α-terpinyl acetate (24.65%) and 1,8-Cineole (14.03%) were identified higher in EC-I than EC-G (18.71% and 10.59% respectively). The high anti-bacterial effects of EC-I are mainly due to these compounds and the other compounds that have antibacterial effects. The compound α-terpinyl acetate is nontoxic and has an effect on neurological disease with anti-inflammatory and anticancer effects [32], similarly, 1,8-Cineole has also been reported as nontoxic [33].

The monoterpene hydrocarbons and oxygenated monoterpenes in the essential oil of different plants possess major antimicrobial, antifungal, and antiviral activities [34]. Our results indicating antibacterial activity against *E. coli* and *P. aeruginosa* are concurrent with those of other studies [20,21]. The cardamom oil was probably active against *P. aeruginosa* and *E. coli* due to the presence of 1,8 cineole and α-terpinyl acetate, which is supported by several investigations [13,34]. Time-kill kinetic studies indicated that essential oil of*E. cardamomum* exhibits bacteriostatic activities against *P. aeruginosa* and *E. coli*, which may be due to the presence of 1,8 cineole, α-terpinyl acetate, and other active antimicrobial volatile agents [35,36,37].

Keeping in view the medicinal use of *E. cardamomum* in multiple gut-related disorders, the essential oils of EC-I (India) and EC-G (Guatemala) were evaluated and compared for their antidiarrheal and gut inhibitory activities through in vivo and in vitro assays. A castor oil-induced diarrhea model was used to study the antidiarrheal effect, whereas isolated rat ileum preparations were used in the in vitro experiments for elucidation of the detailed mechanism [38]. Diarrhea was induced in normal mice by using castor oil, which after hydrolysis into ricinoleic acid, led to evoked spasms in the gut [39]. Pre-administration of both *EC-I* and *EC-G* protected the mice from diarrhea in a dose-dependent manner; however, higher potency was observed with EC-I. After observing the antidiarrheal response, the method described by Palla et al. was followed to test and compare both the samples for antispasmodic effect in vitro in the isolated rat ileum [40]. For this purpose, *EC-I* and *EC-G* cumulative concentrations were added to organ bath after inducing sustained contractions with CCh and high K^+^. Interestingly, both samples demonstrated a dose-dependent complete inhibition of both types of contraction. A critical analysis of the pattern of the inhibitory CRCs of *EC-I* and *EC-G* against CCh and high K^+^-induced contractions indicated that *EC-I* produces relaxation with significantly higher (*p* < 0.05) potency than *EC-G*. The mechanism supposed to be involved in the antispasmodic effect might be the inhibition of a phosphodiesterase (PDE) enzyme [12] and voltage-dependent Ca^++^ channels, because both these mechanisms are involved in smooth muscles relaxation [41,42]. The antidiarrheal effect of EC-I is related to the inhibition of smooth muscle contraction and may be due to the presence of high concentration of the major compound α-terpinyl acetate and 1,8 cineole in this essential oil [43]. The present study elucidates an additional antispasmodic mechanism of cardamom not reported so far, namely the PDE enzyme inhibition. Gilani et al. reported Ca^++^ channel blocking-like mechanisms; however, they did not test it against CCh-induced contractions, which are used to decipher the PDE inhibitory and/or cholinergic mechanisms (REF). Gilani et al. used aqueous methanolic extract, whereas we used essential oil of cardamom [12]. However, our results are also concurrent with those reported by Gilani et al., because they reported that the petroleum ether fraction of cardamom is the most potent in the CCB activity (inhibitory effect at 0.1 mg/mL). We explored the antispasmodic and antidiarrheal effects of cardamom essential oils for the first time, and our findings indicate that the activity of oils varies mainly due to the presence of 1,8 cineole [44]. Compound α-terpinyl acetate inhibited cytochrome P450 enzyme [45]; inhibition of this enzyme may contribute to antidiarrheal effects [46]. In several studies, plants containing major compounds such as sesquiterpenes have also been reported for the biological effects including antimicrobials and antidiarrheal [43,47], so not only the monoterpenes but sesquiterpenes may also contribute to the antidiarrheal effects.

One of the limitations of the current study is that we did not use positive control in our antibacterial assay. For adding further validity, we will direct our future studies to assess the effect of cardamom oil on different pathogenic bacteria involved in gastrointestinal diseases along with using the respective controls including vancomycin and gentamycin for Gram-positive and Gram-negative microbes respectively.

## 4. Materials and Methods

### 4.1. Fruits Samples and Chemicals

Capsules of Indian green cardamom (EC-I) (Emperor Akbar; 250 g) and Guatemalan green cardamom (EC-G) (Al-Othaim; 1 kg) were purchased in January 2019 from the Al-Kharj, Saudi Arabia. Samples were authenticated and kept in the herbarium (Indian: EC-Indian-01-PSAU/3/20 and Guatemala: EC-Guatemala-PSAU/2/20) of the Department of Pharmacognosy, College of Pharmacy, Prince Sattam Bin Abdulaziz University, Al-Kharj, Saudi Arabia. Carbamylcholine (CCh), loperamide, and acetylcholine perchlorate (ACh) were obtained from Sigma Company, St. Louis, MO, USA. Potassium chloride (Sigma Co), calcium chloride, glucose, magnesium sulphate, potassium dihydrogen phosphate, sodium bicarbonate, and sodium chloride (Merck, Darmstadt, Germany) were used as reagents (salts) to prepare physiological buffer solution (Tyrode). All chemicals were of analytical grade, whereas castor oil was purchased from a local pharmacy.

### 4.2. Isolation of Essential Oils

The capsules were ground, and the essential oil was extracted using a Clevenger apparatus. For 3 h, 100 g of each sample powder was extracted, and the percentage yield was calculated after repeating the process thrice. The extracted essential oils were dried over anhydrous Na_2_SO_4_, transferred to an amber-coloured tight vial, labelled as EC-I or EC-G, and stored at 4 °C for further analysis. 

### 4.3. Gas Chromatography–Mass Spectrometry Analysis

The gas chromatography–mass spectrometry (GC–MS) analysis of EC-I and EC-G essential oils was performed using the Shimadzu GC–MS system (TQ-8040, Tokyo, Japan) equipped with auto-sampler (AOC-20i). Analysis of the chemical composition was performed in the ionization mode (70 eV) with a scan time of 0.3 s and *m*/*z* range of 45–400 u. Both the injector and detector temperatures were set at 210 °C. The Rxi-5 MS capillary column (0.25 mm inner diameter, 30 m × 0.25 μm) contained the stationary phase comprising 5% two-phenyl and 95% two-methyl polysiloxane. The column temperature was programmed as follows: Initial oven temperature programmed at 40 °C, held for 3 min; gradually raised to 90 °C at 3 °C/min, held for 4 min; raised to 115 °C at 3 °C/min, held for 10 min; and then increased to 140 °C at 2 °C/min and held for 8 min. Finally, the column temperature was increased to 210 °C at 3 °C/min and held for 5 min. The carrier gas was helium (99.995%) at a constant flow rate of 1 mL/min. The oil identification composition was based on a comparison of their mass spectra and retention time with data of libraries, NIST-14 and NIST-14s (National Institute of Standards and Technologies, Mass Spectra Libraries, Gaithersburg, Maryland, USA) [48]. 

### 4.4. Antibacterial Activity

The antibacterial effect of the EC-I and EC-G oils was tested against two bacterial strains, namely *P. aeruginosa* (ATCC 27853) and *E. coli* (ATCC 35218). The antibacterial activity of the essential oils was assayed using the disc diffusion method [49] with some modification, and each test was repeated thrice. Mueller–Hinton agar (MHA) was used for the antibacterial assay. *P. aeruginosa* and *E. coli* cultures in nutrient broth (HiMedia labs, Mumbai, India) were separately inoculated and grown for 18 h at 37 °C. The suspension of both the organisms was separately diluted with saline (phosphate buffer, pH 7.4) to obtain 1 × 10^6^ colony forming units (CFU/mL) of microbial suspension.

The bacterial inocula were streaked onto an MHA plate by using a sterile swab. A 6-mm sterile disk was impregnated with 10 µL of 5 mg/mL EC-I and EC-G essential oil prepared in 2% DMSO, and 2% dimethyl sulfoxide (DMSO) was used as the negative control. The plates were labelled, and the disks containing essential oil were placed onto the plates and incubated for 18–24 h at 37 °C. The diameter of inhibition around the disk was measured and the mean of three tests was reported as the zone of inhibition. Gentamicin (10 µg) was used as a positive control. 

Different concentrations (4, 2, 1, 0.5, 0.25, and 0.125 mg/mL) of EC-I and EC-G essential oil were prepared in analytical grade 2% DMSO and used for the determination of minimum inhibitory concentration (MIC) through the broth dilution method according to methods described by CLSI 2006 [50]. To each diluted tube, 100 µL inoculum was seeded and the control tubes (no bacterial inoculation) were simultaneously maintained. All tubes were incubated at 37 °C for 24 h and the lowest concentration of the essential oils that produced no visible bacterial growth was recorded. 

### 4.5. Time-Kill Analysis

Time-kill kinetics of essential oil of EC-G and EC-I samples was performed using the method described by Kang et al. [51] with slight modification. Two concentrations equivalent to 1 × MIC (1 mg/mL and 0.5 mg/mL for *E. coli* and *P. aeruginosa*, respectively) and 2 × MIC (2 mg/mL and 1 mg/mL for *E. coli* and *P. aeruginosa*, respectively) of the essential oil were prepared. An inoculum size of 1 × 10^6^ CFU/mL was added and incubated at 37 °C. A total of 1-mm inoculum of the medium was obtained at different time intervals of 0, 2, 4, 8, 12, 18, and 24 h. The colony forming unit (CFU) of the bacterial cells was determined. A negative control containing organisms and DMSO (without essential oil) was also evaluated. The assays were performed in triplicate, and time-kill graphs were constructed by calculating the log CFU/mL of mean colony count against time.

### 4.6. Experimental Animals

*Animals: Wistar* rats (200–250 g; 6–8 weeks old) and Swiss albino mice (25–30 g; 12 weeks or more) of either sex were purchased from the local animal vendors and housed in the animal house of the Barrett Hodgson University (BHU), Karachi, Pakistan. The animals were placed in the transparent plastic rectangular cages (47 × 34 × 18 cm^3^) whose bedding (sawdust) was refreshed every 48 h. Standard temperature (23–25 °C) and a light:dark (12:12) cycle were maintained. During housing, the health status of the animals was also monitored. Standard animal diet was fed, which comprised of flour (380 g/kg), fish meal (170 g/kg), fiber (380 g/kg), molasses (12 g/kg), NaCl (5.8 g/kg), nutrivet L. (2.5 g/kg), potassium metabisulfate (1.2 g/kg), powdered milk (150 g/kg) and vegetable oil (38 g/kg), whereas they were provided with water ad libitum. Before the commencement of the experiments, animals were fasted for a day before the experiment (24 h), however, they were provided with a supply of water. The rats were sacrificed under light anesthesia (using thiopental sodium 70–90 mg/kg; i.p.), followed by cervical dislocation. The guidelines detailed in the National Research Council [52] were followed for all animal-based experiments. The protocols were approved by the Ethical Committee of Research on Animals of the BHU bearing ERC number: BHU-ERC/Pharmacy-001/2020/PI-Dr. Amber Hanif Palla. All results were reported in accordance with the Animal Research: Report of In-vivo Experiments (ARRIVE) guidelines [53].

### 4.7. Gastrointestinal Activities

#### 4.7.1. In Vivo Antidiarrheal Study on Mice

A total of 35 mice were arbitrarily allocated to seven groups with equal numbers of mice in each group. Following 24 h of fasting, mice of the first and second groups were exposed to oral gavage of saline (10 mL/kg) and labelled as sham control and negative control, respectively. After pilot screening for dose selection, the third and fourth groups (test groups) were administered two increasing doses of EC-I (100 and 200 mg/kg; P.O), whereas the fifth and sixth groups were administered EC-G (100 and 200 mg/kg). The last group was administered loperamide (10 mg/kg, P.O.) and labelled as the positive control. Separate cages were assigned to each animal with a blotting sheet on the floor of each cage to know the absence or presence of diarrhea by a blinded observer. After 1 h, all mice except the sham controls were orally exposed to castor oil (10 mL/kg) by using a 1-mL syringe. After 4 h, blotting sheets in all individual cages were inspected for typical diarrheal droppings. Protection was noted in the case of diarrheal drops, as previously reported by Rehman et al., [54].

#### 4.7.2. In Vitro Antispasmodic Activity on Isolated Rat Ileum

The method described by Shah et al., [55] was followed to sacrifice rats and to isolate the ileum, the last part of the small intestine. Briefly, rats were anaesthetized with thiopental sodium (70 mg/kg, given intraperitoneally) and then cervically dislocated by a blow on the head. Following isolation, required segments of the ileum (2–3 cm length) were cleaned from adjacent tissues and fecal material and mounted in a tissue bath (volume 20 mL) that was attached with an isotonic transducer coupled to a digital PowerLab (ML-845) data acquisition system (AD Instruments; Sydney, Australia) and a computer using lab chart software (Version 5.3). A fresh tyrode was filled in 20-mL tissue baths gassed with carbogen, and temperature was set at 37 °C. The composition of Tyrode’s solution (mM) was as follows: KCl, 2.68; NaCl, 136.9; MgCl_2_, 1.05; NaHCO_3_, 11.90; NaH_2_PO_4_, 0.42; CaCl_2_, 1.8; and glucose, 5.55; pH 7.4. Tension of 1 g was applied by rotating the transducer knob clockwise, and the tissues were left for stabilisation for 30 min with multiple exposures to acetylcholine (0.3 µM). After obtaining the stable band in the spontaneous ileal contractions, test samples were added to the bath solution in increasing concentrations, which resulted in the inhibition of the CCh and high K^+^-induced contractions.

### 4.8. Statistics

Results of the antibacterial assay were expressed as the mean of three repeated experiments. Protection from diarrhea was statistically evaluated by comparing all the groups with the saline control group by using Chi square (χ^2^) test. A *p* value of <0.05 was considered statistically significant. Results of the antispasmodic activity assay are expressed as mean ± standard error of mean (SEM). The statistical parameters applied were Student’s *t*-test or two-way ANOVA followed by Bonferroni’s post-test for multiple comparisons of concentration-response curves (CRCs) with control. Graph Pad prism (version 4) was used for regression analysis of CRCs.

## 5. Conclusions

The present findings of GC–MS analysis revealed that α-terpinyl acetate and 1,8 cineole are the major components comparatively higher in EC-I. Monoterpenes were identified as the major components in both the essential oils; however, EC-I was showed to have a higher percentage of monoterpenes than EC-G. Both EC-G and EC-I oils possessed significant antibacterial activity, with EC-I processing more active components than EC-G essential oils. In addition to the antibacterial activity, essential oil of *E. cardamomum* also exhibited antidiarrheal effects along with the antispasmodic activity. Overall, these differences may be due to the presence of different percentages of active and other constituents in the EC-G and EC-I samples. Thus, EC-I exerts more potent antidiarrheal and antispasmodic effects than EC-G. Therefore, present finding delivers a scientific support for the possible future use of E. cardamomum essential oil as an antidiarrheal agent.

## Figures and Tables

**Table 1:** Metabolite identified in the essential oils of Guatemala and Indian *E. cardamomum* using GC-MS.

No	RT	% Area *E. cardamomum*		Compositions
		EC-G	EC-I	
Monoterpenes Hydrocarbons (MTH)				
1	5.436	0.43	0.72	α-Phellandrene
2	5.859	0.09	0.13	Camphene
3	6.296	2.22	3.74	β-Phellandrene
4	6.365	0.66	1.03	Sabinene
5	6.543	2.13	3.46	β-Pinene
6	6.995	1.4	0.06	(+)-2-Carene
7	7.216	1.36	1.77	DL-Limonene
8	7.515	2.01	2.95	β-cis-Ocimene
9	7.738	1.23	1.51	γ-Terpinen
10	8.172	1.22	2.80	α-Terpinene
11	8.595	0.2	-	β-Myrcene
12	10.686	3.21	2.89	β-*trans*-Ocimene
Monoterpenes oxygenated (MTO)				
13	7.478	10.59	14.03	1,8-Cineole
14	7.918	0.81	-	Dihydrocarveol
15	7.921	-	1.25	trans-Sabinenhydrate
16	8.407	5.51	2.63	Linalool
17	8.798	0.79	0.94	α-Terpinenol
18	9.166	0.2	-	*cis*-verbenol
19	9.58	0.96	-	α-Phellandren-8-ol
20	9.737	3.45	3.53	Terpinen-4-ol
21	9.978	3.45	5.11	β-Fenchyl alcohol
22	10.054	0.21	0.49	*n*-Nonyl acetate
23	10.121	-	0.08	*trans*-Pipertiol
24	10.158	0.88	-	Verbenone
25	10.335	1.88	0.31	Citral
26	10.859	4.00	3.55	*cis*-Geraniol (Nerol)
27	11.218	0.35	0.23	Borneyl acetate
28	11.357	0.65	0.12	4-Terpinenyl acetate
29	11.501	-	0.2	Bicyclo [4.1.0]heptan-3-ol, 4,7,7-trimethyl-, (1.alpha.,3.beta.,4.beta.,6.alpha.)-
30	11.607	0.85	0.58	Ocimenyl acetate
31	11.743	1.40	-	Thymol
32	12.389	18.71	24.65	α**-terpinyl acetate
33	12.53	1.93	2.52	*cis*-Geranyl acetate
34	22.385	0.25	-	Myrtanol
Sesquiterpenes hydrocarbons (STH)				
35	12.769	0.44	0.29	β-Eelemene
36	13.335	0.96	0.21	α-Muurolene
37	13.933	0.99	1.58	d-Germacrene
38	14.015	1.28	0.11	Bicyclo[5.2.0]nonane, 2-methylene-4,8,8-trimethyl-4-vinyl-
39	14.06	3.16	1.89	δ-Guaiene
40	14.131	-	1.19	α-selinene
41	14.906	-	0.78	α -caryophyllene
42	15.253	0.91	0.26	γ-Gurjunene
43	17.03	0.37	-	Ledene
44	19.043	0.87	-	Alloaromadendrene
Oxygenated Sesquiterpenes (OST)				
45	13.4	0.49	0.19	*trans*-Caryophyllene oxide
46	13.972	0.79	-	γ-Eudesmol, 10-epi-
47	14.821	5.00	2.29	d-Nerolidol
48	15.684	0.25	-	β-Spathulenol
49	16.01	1.15	-	α-Cadinol
50	16.31	0.41	-	Longifolenaldehyde
51	16.452	0.15	0.06	Aromadendrene oxide-(1)
52	16.526	-	0.12	*trans*-Farnesal
53	16.823	0.3	t	*cis*-Farnesal
54	17.822	0.29	0.14	Farnesyl acetate
55	18.219	0.13	-	Isoaromadendrene epoxide
56	19.381	0.41	-	Costunolide
Diterpenes (DT)				
57	19.951	0.19	-	Cembrene C
58	23.244	0.43	1.08	α-Springene
Oxygenated diterpenes (ODT)				
59	15.108	0.27	-	Kauran-18-al, 17-(acetyloxy)-, (4.beta.)-
60	21.797	0.14	-	Thunbergol
Non-terpenes				
61	6.42	0.18	t	6-Methyl-5-hepten-2-one
62	6.775	0.31	0.29	Octanal
63	8.222	0.14	0.14	Benzene, 2-ethenyl-1,3-dimethyl-
64	9.32	0.16	0.16	Sabine ketone
65	9.393	0.13	0.47	1,2-Dimethyl-3,5-divinylcyclohexane
66	9.842	-	0.25	*cis*-4-Decenal
67	12.674	0.18	-	Cyclodecene
68	12.679	-	1.01	9,17-Octadecadienal
69	13.01	0.12	0.23	1-Decanol acetate
70	15.088	-	0.31	5,7-Dodecadiene, (*Z*,*Z*)
71	15.534	0.42	-	7-Heptadecyne, 1-chloro-
72	20.153	0.27	0.44	Tetracosamethyl-cyclododecasiloxane
73	20.827	0.91	0.48	Cyclodeca-cyclotetradecene, 14,15-didehydro-
74	22.557	1.94	1.69	Eicosamethylcyclodecasiloxane

RT: Retention time (min) and percentage area based on available libraries (e.g., NIST or Wiley). Others compounds: a: Ketone, b: Aldehyde, c: Ester, d: Alkane, e: Alkene, f: Cycloalkane, g: Benzene, h: Halogen, i: nor isoprenoids, j: siloxane, (-): not identified, (t): trace.

**Table 2:** Class of terpene identified in the essential oils obtained from Guatemala and Indian *E. cardamomum*.

Terpenes	EC-G		EC-I	
	No. of Compounds	% Area	No. of Compounds	% Area
Monoterpenes Hydrocarbons	12	16.16	11	21.06
Oxygenated monoterpenes	19	56.87	17	60.22
Total Monoterpenes	31	73.03%	28	81.28%
Sesquiterpenes Hydrocarbons	8	8.98	9	6.38
Oxygenated Sesquiterpenes	11	9.37	6	2.89
Total Sesquiterpenes	19	18.35%	14	9.27%
Diterpenes hydrocarbons	2	0.62	1	1.08
Oxygenated diterpenes	2	0.41	0	0
Total Diterpenes	4	1.03%	1	1.08%
Non terpenes	9	6.41%	10	6.09
Total (75)	63	98.82	53	97.72

**Table 3:** Antimicrobial activity of the essential oils obtained from EC-G and EC-I.

Microorganism	EC-G		EC-I		Gentamycin (10 µg)
	ZOI (mm)	MIC (mg/mL)	ZOI (mm)	MIC (mg/mL)	ZOI (mm)
*P. aeruginosa*	12.33 ± 0.27	0.50	16.66 ± 0.47	0.25	22.70 ± 0.21
*E. coli*	10.13 ± 0.23	1.00	14.40 ± 0.10	0.50	19.67 ± 0.15

**Table 4:** Comparative antidiarrheal activities of the extracted essential oil of *Elettaria cardamomum* of Indian (EC-I) and Guatemala (EC-G) on castor oil (10 mL/kg)-induced diarrhea in mice.

Treatment (p.o.), Dose (mg/kg)	No. of Mice with Diarrhea	% Protection
Saline (10 mL/kg) + Castor oil	5/5	0
EC-I (100 mg/kg) + Castor oil	3 */5	40
EC-I (200 mg/kg) + Castor oil	1 */5	80
EC-G (100 mg/kg) + Castor oil	4/5	20
EC-G (200 mg/kg) + Castor oil	2 */5	60
Loperamide (10 mg/kg) + Castor oil	0 **/5	100

* *p* < 0.05 and ** *p* < 0.01 vs. Saline + Castor oil treated group (χ^2^-test).

## Data Availability

Data supporting the findings of this study are available within the article.

## Sample Availability

Fruits samples are available at Department of Pharmacognosy, College of Pharmacy, PSAU-Al-Kharj, KSA.
